# Analysis of agreement between cardiac risk stratification protocols applied to participants of a center for cardiac rehabilitation

**DOI:** 10.1590/bjpt-rbf.2014.0159

**Published:** 2016-04-01

**Authors:** Ana A. S. Santos, Anne K. F. Silva, Franciele M. Vanderlei, Diego G. D. Christofaro, Aline F. L. Gonçalves, Luiz C. M. Vanderlei

**Affiliations:** 1 Departamento de Fisioterapia, Faculdade de Ciências e Tecnologia, Universidade Estadual Paulista (UNESP), Presidente Prudente, SP, Brazil; 2 Departamento de Educação Física, Faculdade de Ciências e Tecnologia, UNESP, Presidente Prudente, SP, Brazil

**Keywords:** cardiology, exercise, protocols, rehabilitation

## Abstract

**Background:**

Cardiac risk stratification is related to the risk of the occurrence of events induced by exercise. Despite the existence of several protocols to calculate risk stratification, studies indicating that there is similarity between these protocols are still unknown.

**Objective:**

To evaluate the agreement between the existing protocols on cardiac risk rating in cardiac patients.

**Method:**

The records of 50 patients from a cardiac rehabilitation program were analyzed, from which the following information was extracted: age, sex, weight, height, clinical diagnosis, medical history, risk factors, associated diseases, and the results from the most recent laboratory and complementary tests performed. This information was used for risk stratification of the patients in the protocols of the American College of Sports Medicine, the Brazilian Society of Cardiology, the American Heart Association, the protocol designed by Frederic J. Pashkow, the American Association of Cardiovascular and Pulmonary Rehabilitation, the Société Française de Cardiologie, and the Sociedad Española de Cardiología. Descriptive statistics were used to characterize the sample and the analysis of agreement between the protocols was calculated using the Kappa coefficient. Differences were considered with a significance level of 5%.

**Results:**

Of the 21 analyses of agreement, 12 were considered significant between the protocols used for risk classification, with nine classified as moderate and three as low. No agreements were classified as excellent. Different proportions were observed in each risk category, with significant differences between the protocols for all risk categories.

**Conclusion:**

The agreements between the protocols were considered low and moderate and the risk proportions differed between protocols.

## Bullet points

- Studies indicating similarity between cardiac risk stratification protocols are important in clinical practice.
- Most protocols for cardiac risk stratification present low or moderate agreements.
- The protocols have shown good applicability to most patients.

## Introduction

Cardiovascular diseases (CVD) are the leading cause of death in most countries, including Brazil, accounting for about 20% of all deaths in individuals over 30 years of age^1^ ^,^ ^2^. In addition to the high mortality rate, these diseases may be responsible for physical disability and contribute significantly to increased spending on health^1^. This scenario demonstrates the need for effective interventions, of which cardiac rehabilitation (CR) seems to be one example. According to the World Health Organization^3^, CR is the range of proposed activities to ensure better living conditions for an individual with heart disease, as well as contributing to the improvement in functional capacity^4^, having an important role in preventing cardiovascular events and reducing mortality from these conditions^5^.

The central idea for CR is to perform exercise, the prescription of wich should be made on an individual basis in order to provide beneficial effects and safety during performance^6^ ^,^ ^7^. The first step for the prescription is to conduct a thorough evaluation of the clinical and functional status of the patient which, among other things, provides a patient risk stratification, related to the possible risk of adverse events induced by exercise during performance of the CR program^8^, guiding the form and intensity of the work to be performed with the cardiac individual.

In a literature review conducted by our group^7^, eight risk stratification protocols were found, developed, and validated by various national and international entities, devised for the participation of individuals in exercise programs and CR.

The criteria for stratification consider factors associated with an increased risk of morbidity and mortality during physical exercise, and based on these criteria, the individual is usually classified as low, moderate, or high risk^7^ ^,^ ^9^. In addition to the knowledge of the risk level, stratification provides information for the proper direction of the patient throughout the CR process and planning of the program^10^, aiding the professional to determine the appropriate level of monitoring in accordance with the risk level of the patient^8^.

However, the existence of multiple risk stratification protocols may hamper the selection of the most suitable to be used during the CR process. A search in the literature found no studies evaluating the similarity between the risk stratification protocols, demonstrating gaps in the literature that raise the following questions: Would an individual be classified in the same risk level in different protocols? Are there agreements between the risk ratings used in the protocols? If so, which ones can be considered similar and which differ? This information may contribute to researchers and clinicians who act in CR programs, giving safer direction for adopted behaviors and exercise prescription with cardiac patients and even identifying differences between specific protocols.

One of the few studies that compared protocols was carried out by Paul-Labrador et al.^11^ that evaluated American patients and found that the ability of the guidelines (American Association of Cardiovascular and Pulmonary Rehabilitation [AACVPR]^9^, American Heart Association [AHA]^12^, American College of Cardiology [ACC]^13^, and American College of Physicians [ACP]^14^) to predict complications in patients at high risk was low.

In this context, the aim of this study was to evaluate the level of agreement between existing protocols on the cardiac risk score in heart disease. It was hypothesized that despite the differences between the protocols, there would be agreement between them regarding the prediction of the risk of developing complications during exercise.

## Method

This is a descriptive/analytical cohort study, transversal, with a restropective characteristic, developed from data drawn from 50 medical records of individuals seen in an outpatient exercise program for patients with cardiovascular disorders, between April and May 2014, with no restrictions concerning age or gender.

These patients received information about the objectives and procedures of the study and signed a consent form authorizing the use of their data. All procedures were approved by the Research Ethics Committee of Universidade Estadual Paulista Júlio de Mesquita Filho (UNESP), Presidente Prudente, SP, Brazil, under protocol number 792.373 of 05/09/14.

### Data collection

The patient records were analyzed and the following information extracted: age, gender, weight, height, clinical diagnosis for which the patient was referred to the heart disease unit, medical history, risk factors (RF) for the development of CVD, associated diseases, and the results of recent laboratory tests and complementary tests related to the evaluation of the cardiovascular system (exercise testing, echocardiography, Holter monitoring, cardiac catheterization, echo-stress, myocardial scintigraphy, electrocardiogram, and coronary angiogram). This information was tabulated and subsequently used for risk stratification of patients using the different protocols evaluated in this study. A single evaluator performed all data collection.

### Characterization of participants

The characterization was based on information regarding age, weight, height, and clinical diagnosis. Body mass index (BMI) was calculated from weight and height using the formula: body weight (kg)/height^2^(m) to determine the obesity risk factor according to the criteria of the Brazilian Obesity Guidelines – 3^rd^ edition^15^.

### Clinical diagnosis

The main clinical diagnosis was considered as the diagnosis that led to referral to the unit. Patients with coronary artery disease (CAD) were divided into groups according to the procedure performed: coronary artery bypass grafting (CABG), angioplasty with stent placement, CABG and stent, or conservative treatment. Patients with heart failure (HF) were also subdivided according to the New York Heart Association (NYHA) Functional Classification^16^. Patients without the presence of diagnosed heart disease were allocated to the preventive group.

### Medical history

The following information was extracted from the medical records: the presence and number of cardiopulmonary arrests, number of days of hospitalization, complications during hospital stay or after performing an invasive procedure, and current complications.

### Risk factors

The information contained in the medical records was analyzed for the presence of the following risk factors (RF): sex/age (men over the age of 45 years and women over 55), family history (considering the presence of CVD in first-degree relatives), hypertension, dyslipidemia, and smoking. For the obesity risk factor, the value obtained in the calculation of BMI was used, considering patients obese with a BMI≥30 kg/m^2 15^.

### Associated diseases

Associated diseases were considered as any musculoskeletal, neurological, pulmonary, or metabolic dysfunction, with the data obtained from the medical records.

### Laboratory tests and complementary tests

The most recent exams recorded in the medical records were analyzed, regardless of the results contained in these exams. Blood glucose, triglycerides, total cholesterol, HDL-cholesterol, and LDL-cholesterol were obtained from the laboratory records. Only complementary tests that assessed the cardiovascular system were used. The results for patients who had undergone Holter monitoring were classified according to Lown and Wolf^17^, which takes into account ventricular premature beats for determining the appropriate risk class, based on the frequency and severity with which they appear.

### Stratification of cardiac risk

For risk stratification, we used the following protocols described in the study of Silva et al.^7^: American College of Sports Medicine (ACSM)^18^, Brazilian Society of Cardiology (BSC)^19^, AHA^12^, the protocol designed by Pashkow^10^, AACVPR^9^, French Society of Cardiology (FSC)^20^, and Spanish Society of Cardiology (SSC)^21^. Risk stratification was performed on each protocol using the same complementary tests for each patient. In all protocols, the patients were classified as low, moderate, or high risk, and the presence of any characteristics in a higher cardiac risk band ranked the individual in that category.

The guidelines of the ACSM^18^ were used as a basis for classifying information on age, health status, symptoms, and RF. The BSC protocol^19^ is based mainly on maximal exercise test results to identify myocardial ischemia, ventricular dysfunction, cardiac arrhythmias, and atrioventricular conduction disturbances.

Unlike previous guidelines, AHA^12^ classifies patients into risk classes (A, B, C, and D) and takes into account the presence of symptoms or heart disease, RF, and findings of the exercise test. For this protocol, patients classified as class A were considered low-risk patients, class B as moderate risk, and class C as high risk. According to this classification, patients in class D should not participate in a CR program^12^, therefore this class was not included in the study. In 1993, Pashkow^10^ developed a model of risk stratification based on important guidelines at the time as well as a new means of risk identification that bases stratification on the results of tests such as the progressive stress test, electrocardiogram, and echocardiogram.

The guidelines of the AACVPR^9^ stratifies patients based mainly on the findings of the ergometric test. According to these guidelines, patients who do not undergo this test before entering the program or those with undiagnosed exercise tests could be categorized inappropriately and therefore risk stratification should be approached with caution.

The FSC protocol^20^ is adapted from the recommendations of the European Society of Cardiology, and the AACVPR and is based mainly on the findings of the stress test and echocardiogram for the classification of the patient. The SSC protocol^21^, published in the Practice Guidelines on Cardiovascular Prevention and Rehabilitation, stratifies patients using clinical data and findings of examinations, especially echocardiography and the ergometric test.

The protocols are aimed at the participation of individuals in exercise programs and/or CR and covers patients diagnosed with coronary artery disease, particularly post-acute myocardial infarction, although they are expandable to all CVD^9^ ^,^ ^10^ ^,^ ^12^ ^,^ ^19^ ^-^ ^21^ and can also be applied to healthy individuals that present RF^18^.

### Statistical analysis

Data normality was tested by the Shapiro-Wilk test. To characterize the sample, descriptive statistics were used, with the results presented as mean, standard deviation, absolute and relative values. Comparison of risk ratios (low, moderate, or high risk) between the protocols was performed by applying the chi-square tests. To analyze the agreement among the protocols, the Kappa index was used. The agreement was considered weak for k values below 0.40, moderate for values between 0.40 and 0.75, and excellent for k values greater than 0.75. Differences were considered significant if p<0.05. The software used was SPSS version 15.0.

## Results

Table 1 presents the characteristics of the study population. Of the patients who had a main diagnosis of CAD, 37.0% (n=10) underwent angioplasty with stent placement, 33.3% (n=9) CABG, 7.4% (n=2) both stent and CABG, and 22.2% (n=6) performed conservative treatment. Among the patients diagnosed with IC it was observed that 22.2% (n=2) were classified in functional class I and 77.8% (n=7) in functional class II, according to the NYHA Functional Classification^16^.

**Table 1:** Characteristics of the study population (n=50).

**Characteristics**	**Values**
Sex (M/F)	33 (66%) / 17 (34%)
Age (years)	65.50±9.56 [39 – 81]
Weight (Kg)	79.30±13.09 [54.00 – 105.50]
Height (m)	1.65±0.08 [1.46 – 1.78]
BMI (Kg/m^2^)	29.13±4.22 [20.68 – 38.25]
Principal Diagnosis	
Coronary Insufficiency	27 (54%)
Heart Failure	09 (18%)
Constrictive Pericarditis	01 (2%)
Atrial Fibrillation	01 (2%)
Valvulopathy	01 (2%)
Coronary Artery Dissection	01 (2%)
AMI	03 (6%)
Cardiovascular risk factors	07 (14%)

Values expressed as mean±standard deviation [Minimum value – Maximum value] or in absolute values and percentages. M: male; F: female; Kg: kilogram; m: meters; BMI: body mass index; m^2^: meters squared; AMI: acute myocardial infarction.

Table 2 shows the complementary tests present in the records. The most commonly used were laboratory examinations, echocardiography, and an ergometric test.

**Table 2:** Absolute values and percentage of complementary tests found in the patient’s medical records.

**Exam**	**Values**
Coronary artery angiography	02 (4%)
Myocardial scintigraphy	10 (20%)
Catheterization	22 (44%)
Echocardiography	35 (70%)
Echo-stress	01 (2%)
Electrocardiogram	21 (42%)
Laboratory exam	43 (86%)
Exercise test	35 (70%)
Holter monitoring	17 (34%)

Table 3 shows the percentage distribution of risk classification for each protocol. Different proportions were observed in each category of risk stratification among the evaluated protocols, with less accurate proportions between the protocols of the AHA and ACSM. It should be noted that there were statistically significant differences between the protocols for all risk categories.

**Table 3:** Absolute values and percentage of the risk classification of patients according to the protocols analyzed.

**Protocols**	**Low risk**	**Moderate risk**	**High risk**	**P-value**
ACSM	0 (0.0%)	5 (10.0%)	45 (90.0%)	≤0.001
BSC	19 (40.4%)	16 (34.1%)	12 (25.5%)	0.009
AHA	7 (14.0%)	37 (78.6%)	3 (6.4%)	≤0.001
Pashkow	5 (10.7%)	27 (57.4%)	15 (31.9%)	≤0.001
AACVPR	18 (37.5%)	6 (12.5%)	24 (50.0%)	≤0.001
FSC	17 (36.2%)	11 (23.4%)	19 (40.4%)	≤0.001
SSC	18 (38.4%)	13 (27.6%)	16 (34.0%)	≤0.001

Values expressed in absolute values and percentages. ACSM: American College of Sports Medicine; BSC: Brazilian Society of Cardiology; AHA: American Heart Association; AACVPR: American Association of Cardiovascular and Pulmonary Rehabilitation; FSC: French Society of Cardiology; SSC: Spanish Society of Cardiology.

The agreement between risk classifications obtained from the protocols can be seen in Table 4. Of the 21 agreements obtained, 12 were considered significant, nine classified as moderate, and three as low, according to the Kappa coefficient. A significant p value was observed in all moderate agreements.

**Table 4:** Agreement of risk classification between the protocols used.

**Protocols**	***K***	***P-value***
ACSM vs BSC	0.06	0.241
ACSM vs AHA	0.01	0.537
ACSM vs Pashkow	0.04	0.417
ACSM vs AACVPR	0.00	0.999
ACSM vs FSC	0.02	0.683
ACSM vs SSC	0.02	0.690
BSC vs AHA	0.04	0.711
BSC vs Pashkow	0.74	≤0.001
BSC vs AACVPR	0.52	≤0.001
BSC vs FSC	0.57	≤0.001
BSC vs SSC	0.39	0.006
AHA vs Pashkow	0.00	0.931
AHA vs AACVPR	0.04	0.550
AHA vs FSC	0.19	0.028
AHA vs SSC	0.22	0.016
Pashkow vs AACVPR	0.57	≤0.001
Pashkow vs FSC	0.72	≤0.001
Pashkow vs SSC	0.56	≤0.001
AACVPR vs FSC	0.65	≤0.001
AACVPR vs SSC	0.44	0.001
FSC vs SSC	0.68	≤0.001

ACSM: American College of Sports Medicine; BSC: Brazilian Society of Cardiology; AHA: American Heart Association; AACVPR: American Association of Cardiovascular and Pulmonary Rehabilitation; FSC: French Society of Cardiology; SSC: Spanish Society of Cardiology.

The agreement table also shows that the ACSM protocol had no agreement with any of the analyzed protocols and that the AHA protocol presented agreement only with the FSC and SSC, though the agreement was low. Low agreement was also observed between the BSC and SSC protocols, and the highest agreement was found between the BSC protocol and the Pashkow protocol.

## Discussion

This study aimed to evaluate the level of agreement between existing risk stratification protocols in individuals who regularly attended a CR program. According to the results observed in this study, there were only 12 agreements between the evaluated protocols, which were classified as low or moderate, without any agreement considered excellent. Different proportions in each category of risk stratification were observed among the evaluated protocols, this ratio being less accurate between the AHA and ACSM.

This is the first study to evaluate the existence of agreements between protocols developed to stratify the risk of individuals in exercise programs and CR. Different proportions in each category of risk stratification were observed in the majority of the evaluated protocols. In the ACSM protocol, 90% of patients were classified as high risk in contrast to the AHA, which ranked only 6.4% of these patients in the same risk category. One of the reasons for this discrepancy is the fact that the ACSM protocol is the only one that uses the presence of cardiovascular, metabolic or pulmonary disease as criteria for classifying the individual as high risk^7^ ^,^ ^18^ and the majority of the sample (45 individuals) comprised individuals with these disorders, which contributed to the high prevalence of high-risk individuals in this protocol.

Depending on the stratification criteria adopted by the protocols, not all were able to stratify all individuals in the sample. In the BSC, FSC, SSC, and Pashkow protocols, it was not possible to stratify three patients who did not have complementary tests or information that allowed allocation into one of the stratification criteria. In the AACVPR protocol, only one of these three patients was stratified, because although the patient had no complementary test, their clinical condition satisfied one of the stratification criteria of this protocol.

In the AHA protocol, three patients could not be stratified. Two patients had complementary tests, but basic conditions (atrial fibrillation and constrictive pericarditis) did not meet any of the criteria for determining the risk class, and one patient had no complementary tests to determine the risk class, although the underlying disease was among the criteria.

The above data highlight the importance of complementary tests in the risk stratification process of these patients. Complementary tests related to the evaluation of the cardiovascular system were present in 94% of the analyzed records. Among the patients with complementary tests, only one had not undergone the ergometric test or echocardiography, which are examinations used as stratification criteria for the majority of protocols^9^ ^,^ ^10^ ^,^ ^12^ ^,^ ^19^ ^-^ ^21^, however the patient had an echo-stress, which enables not only the evaluation of contractile function but also the verification of the presence of ischemia with stress. The ergometric test and echocardiogram are very important in the stratification process, since the protocols that use them consider their findings in more than one level and in many cases are the defining tests for the risk presented by the patient.

Regarding the agreement between the protocols, it was observed that the risk classification of the ACSM protocol did not agree with any of the other protocols used, which is related to the fact that this protocol uses the clinical history of the patient as the criterion for stratifying, without the use of complementary tests.

Due to the low number of similarities in the risk ranges between the AHA protocol and other protocols, this protocol did not agree with four of the six guidelines and the two agreements that were found, with the protocols of the FSC and the SSC, were classified as low according to the Kappa coefficient (K<0.40).

The FSC and Pashkow were the protocols that obtained the highest number of moderate agreements (each with four protocols) with other protocols. The highest agreement was observed between the BSC and Pashkow protocols (K=0.74) and it was close to the value considered as excellent (K>0.75). These protocols have similarities regarding the criteria for risk classification that may explain, at least in part, the agreement found. In addition, the criteria use the most prevalent examinations in the study sample.

The majority of protocols examined in this study are based on the findings of complementary tests for cardiac risk classification^9^ ^,^ ^10^ ^,^ ^12^ ^,^ ^19^ ^-^ ^21^. Among the tests used for risk stratification of heart patients, the ergometric test stands out due to its well-established methodology^22^. This test is used to identify myocardial ischemia and arrhythmia induced by exercise and it provides the value of the metabolic equivalent (MET), which is utilized in all protocols that use complementary tests as one of the main references to determine the risk level^7^. Despite its importance, 30% of the patients had no ergometric test in their medical records.

Echocardiographic data are also used by six of the seven protocols, and ejection fraction is a reference in five protocols^9^ ^,^ ^12^ ^,^ ^19^ ^-^ ^21^. The value of the ejection fraction obtained by echocardiography is considered a risk predictor^23^. As well as an ergometric test, 30% of the patients included in the study did not have an echocardiogram in their records.

Some factors that contributed to the disagreements between the protocols were: i) a number of the protocols^9^ ^,^ ^10^ ^,^ ^19^ ^-^ ^21^ are directed mainly at patients who suffered acute myocardial infarction; ii) many of the tests used have discrepancies between the protocols, i.e. not all information concerning the ergometric test and echocardiogram was used to characterize the individual in relation to the risk classification.

This study has some limitations that should be pointed out. The fact that some records contained incomplete medical history without some details necessary to some protocols, such as the presence of complications during hospitalization, reinfarction, and the occurrence of cardiac arrest, may have produced errors in the stratification of patients in some protocols (AACVPR^9^, AHA^12^, FSC^20^, and SSC^21^). It is suggested that further studies follow these patients for a period of time, as done by Paul-Labrador et al.^11^ to verify if the protocols are able to predict the development of complications. Another factor to be considered is the lack of a cutoff score in all protocols, since such information could be used in other analyses to verify the agreement between the protocols, such as the intraclass correlation coefficient and Bland-Altman plot.

The results of this study do not allow us to determine the best protocol to be used, however it can be stated that the ACSM protocol is the most suitable for patients who have no complementary tests. All of the other protocols can be used in patients who have complementary tests, however according to the tests they have and the patient's clinical history, some protocols may be more suitable for assessing the risk of cardiac events during exercise.

The SSC protocol^21^ is the only one that takes into account the occurrence of reinfarction among its criteria for classification and a history of cardiac arrest is a criterion used by the AACVPR^9^, AHA^12^, and FSC protocols^20^. The FSC protocol^20^ also differs from the others in the use of the Lown classification in their criteria, a protocol indicated for patients who have the Holter monitoring among their complementary tests. Myocardial scintigraphy information is used by the SSC^21^, BSC^19^, FSC^20^, and Pashkow^10^ protocols, with the last two also addressing echo-stress test results.

The AHA protocol^12^, despite being introduced as an extensive method for the stratification of patients, does not possess all existing cardiac diseases in its criteria and thus is not feasible to determine the risk for all patients included in a CR program. In contrast, this is the only protocol that takes into account the NYHA Functional Classification^16^ as one of the stratification criteria.

The majority of the evaluated protocols presented low or moderate agreement. However, despite their limitations, all protocols showed good applicability for the cardiac risk assessment of the majority of the patients, which is important for professionals acting in the CR area.

## Conclusion

Based on the findings of the present, it can be concluded that the agreements between cardiac risk classification protocols in cardiac patients undergoing a CR program were considered low to moderate. Furthermore, the risk ratios differed between protocols according to each category of risk stratification.
